# Stone Paper as a New Substrate to Fabricate Flexible Screen-Printed Electrodes for the Electrochemical Detection of Dopamine

**DOI:** 10.3390/s20123609

**Published:** 2020-06-26

**Authors:** Codruta Varodi, Florina Pogacean, Marin Gheorghe, Valentin Mirel, Maria Coros, Lucian Barbu-Tudoran, Raluca-Ioana Stefan-van Staden, Stela Pruneanu

**Affiliations:** 1 National Institute for Research and Development of Isotopic and Molecular Technologies, Donat Street, No. 67–103, RO, 400293 Cluj-Napoca, Romania; codruta.varodi@itim-cj.ro (C.V.); florina.pogacean@itim-cj.ro (F.P.); valentin.mirel@itim-cj.ro (V.M.); maria.coros@itim-cj.ro (M.C.); lucianbarbu@yahoo.com (L.B.-T.); 2 NANOM MEMS srl, G. Cosbuc Street, No. 9, RO, 505400 Rasnov, Brasov, Romania; maringhe@nanom-mems.com; 3 Laboratory of Electrochemistry and PATLAB, National Institute of Research for Electrochemistry and Condensed Matter, 202 Splaiul Independentei Street, 060021 Bucharest-6, Romania; ralucavanstaden@gmail.com; 4 Faculty of Applied Chemistry and Material Science, Politehnica University of Bucharest, 060042 Bucharest, Romania

**Keywords:** flexible screen-printed electrodes, stone paper, gold nanoparticles, dopamine detection

## Abstract

Flexible screen-printed electrodes (HP) were fabricated on stone paper substrate and amperometrically modified with gold nanoparticles (HP-AuNPs). The modified electrode displayed improved electronic transport properties, reflected in a low charge-transfer resistance (1220 Ω) and high apparent heterogeneous electron transfer rate constant (1.94 × 10^−3^ cm/s). The voltammetric detection of dopamine (DA) was tested with HP and HP-AuNPs electrodes in standard laboratory solutions (pH 6 phosphate-buffered saline (PBS)) containing various concentrations of analyte (10^−7^–10^−3^ M). As expected, the modified electrode exhibits superior performances in terms of linear range (10^−7^–10^−3^ M) and limit of detection (3 × 10^−8^ M), in comparison with bare HP. The determination of DA was tested with HP-AuNPs in spiked artificial urine and in pharmaceutical drug solution (ZENTIVA) that contained dopamine hydrochloride (5 mg/mL). The results obtained indicated a very good DA determination in artificial urine without significant matrix effects. In the case of the pharmaceutical drug solution, the DA determination was affected by the interfering species present in the vial, such as sodium metabisulfite, maleic acid, sodium chloride, and propylene glycol.

## 1. Introduction

In the last few decades there was growing research into the development of a new generation of biodegradable electronics [1,2]. The interest was directed towards non-toxic materials, eco-friendly solvents and reagents, with a design that minimizes the amount of waste, considering each step of the device’s life-time, from production to disposal. The usage of renewable and waste-based materials as substrates for screen-printed electrodes was described by Moro G et. al., in their recently published paper [3]. Other types of material used as electrode substrates are polyvinyl chloride (PVC), ceramics and paper. The trend of paper-based sensor publications has increased exponentially in the last few years [4,5]. Nowadays, the paper is a material with a high rate of utilization as electrodes’ substrate, due to its biodegradability, flexibility, low price, commercial availability and easiness to use. The main disadvantages are related to deforestation and to the large water consumed in the manufacturing process.

Stone paper or mineral paper is another kind of paper, very attractive for flexible device applications. It is a composite material made of calcium carbonate and a non-toxic resin, high-density polyethylene (HDPE). Stone paper has a number of advantages over traditional paper, being water- and grease-proof, tearing with difficulty due to a latex-like texture, and generally being more durable than normal paper [6]. In addition, the production of paper from stone offers significant environmental benefits, such as: no deforestation, no water, bleach or acid used in the production steps. The manufacturing process produces less effluent and consumes half as much energy as traditional paper-making. It may be recycled with plastics or remade into rich mineral paper again, and is not biodegradable but is photo-degradable under suitable conditions [7].

As a kind of catecholamine, dopamine (DA) has been gaining increasing focus in the field of clinical research, therapeutically for its effects on the kidneys (promoting natriuresis) and on the cardiac muscle (increasing the cardiac output) [8]. It is a neurotransmitter synthesized from tyrosine and is measured in the investigation of suspected catecholamine-secreting tumors. It is involved in cognitive function and a long-term excessive production of DA can lead to irritability, uncontrollable emotions, fast nerve reflexes, and extreme hyperactivity, but an extremely low level can cause neurological disorders including Parkinson’s disease and schizophrenia [9].

The electrochemical detection of DA is highly advantageous over other conventional techniques (e.g., optical, fluorometry, chromatography and mass spectrometry [10,11,12,13] with good sensitivity, selectivity, and simplicity. Several papers are reported in the literature with respect to the detection of DA using various electrodes [14,15] including exfoliated flexible graphite paper [16], carbon nanotube modified screen-printed electrodes [17], microfluidic paper-based analytical devices modified with graphene-based nanomaterials [18], nanogold-modified screen printed carbon electrodes [19], paper-based electrode with carbon conductive ink [20] or ordered mesoporous carbon covered carbonized silk fabrics [21]. The different characteristics of each type of electrode determine its own advantages and inconveniences.

In this work, the stone paper is proposed as a new substrate to fabricate flexible screen-printed electrodes employed for the electrochemical detection of dopamine. The stone paper is cheaper, more flexible and easier to print compared with other commonly used substrates, such as polyethylene terephthalate (PET), polyimide and polydimethylsiloxane (PDMS). The affinity of various NPs (gold, silver or carbon-based materials) is very high, due to its porous morphology. The modification of the stone paper substrate can be realized either by screen-printing or by drop-casting the desired material. In our case, the screen-printing was chosen to fabricate the three-electrode system, necessary for electrochemical measurements. The working electrode was further modified with gold nanoparticles, to improve its electro-catalytic properties.

## 2. Materials and Methods

### 2.1. Chemical and Reagents

All chemicals, including CaCl_2_·H_2_O (Alfa Aesar, Germany), NaCl, Na_2_SO_4_, KH_2_PO_4_, KCl, NH_4_Cl, anhydrous creatinine (C_4_H_7_ON_3_, ≥98%), urea (CH_4_N_2_O, 99.0%) (from Sigma-Aldrich, Germany) were used for the preparation of artificial urine, without further purification. Potassium ferrocyanide K_4_[Fe(CN)_6_] and KCl were purchased from Sigma-Aldrich, Germany. Dopamine hydrochloride was purchased from Alfa-Aesar (Germany). Double-distilled water was obtained with a Fistreem Cyclon water purification system (UK) and used to prepare all the solutions. Stone paper was purchase from MIQUELRIUS (Spain) and used as substrate for screen-printed electrode fabrication.

### 2.2. Apparatus

A Potentiostat/Galvanostat Instrument (PGSTAT-302N, Metrohm-Autolab B.V., Utrecht, The Netherlands) was used for the electrochemical measurements (cyclic voltammetry—CV; linear sweep voltammetry—LSV and electrochemical impedance spectroscopy—EIS). The impedance spectra were recorded over 0.1–10^6^ Hz range (10 mV amplitude signal) and the experimental data were fitted using Nova 1.11 software.

Scanning electron microscopy (SEM) images and energy-dispersive X-ray spectroscopy (EDS) analyses of stone paper electrodes were registered with a Hitachi SU8230 (Japan) high-resolution scanning electron microscope equipped with a cold field emission gun.

Fourier transform infrared (FTIR), Raman and Brunauer–Emmett–Teller (BET) analyses were employed to characterize the graphite powder, used to screen-print the flexible substrate.

The FTIR spectrum was recorded with a Bruker Tensor-II spectrometer (Germany) using the KBr pellet technique (4000–400 cm^−1^ range).

Raman spectrum was collected with a JASCO-NRS 3300 Spectrophotometer (USA) equipped with a charge-coupled device (CCD) detector (−68 °C), using a 600 L/mm grid. The incident laser beam (1 mm^2^) was focused with an Olympus Microscope and 100x objective. The excitation was performed with an Ar-ion laser (514 nm) with the power at the sample surface of 1.3 mW.

Evaluation of specific surface area and pore size distribution were performed following the standard BET procedure by N_2_ adsorption–desorption at −196 °C (Sorptomatic 1990, Thermo Electron, Italy). Prior to N_2_ adsorption, graphite sample was degassed under vacuum at 200 °C, for 4 h.

### 2.3. Fabrication of Flexible Screen-Printed Electrodes on Stone Paper Substrate

Three special inks (based on silver flake, graphite powders and, respectively, on dielectric material) with low temperature of processing (maximum 80 °C) were developed by NANOM MEMS srl. A three-electrode system was printed with a semiautomatic screen-printer (LC-TA-250 Model) using stone paper as substrate and was denoted HP.

**Figure 1:**
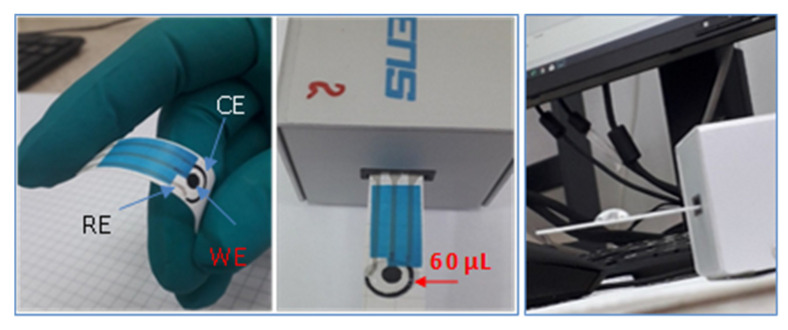
Optical images of the flexible screen-printed electrodes.

The flexible electrode can be easily connected to a laboratory potentiostat or mini-potentiostat for on-site analysis (Figure 1).

Generally, Au or Pt nanoparticles are used to modify various surfaces, but in most cases the nanoparticles are obtained from the corresponding metal salt solution (e.g., HAuCl_4_ or H_2_PtCl_6_) by reduction at high temperature (100 °C) with sodium citrate. In our case, we developed an easier method where gold ions from HAuCl_4_ solution were electrochemically reduced to nanoparticles (AuNPs). Hence, the screen-printed electrodes were modified with AuNPs using the chronoamperometric method. The working electrode was polarized for 120 s at –0.2 V vs. silver pseudo-reference electrode, in 0.5 M H_2_SO_4_ + 1 mM HAuCl_4_ solution. The innovativeness of the method consists in the very short time (120 s) necessary to modify the flexible electrode. The Au-NPs modified HP electrode was denoted HP-AuNPs. A volume of 60 μL solution was typically necessary to record the measurement with HP or HP-AuNPs electrode (Figure 1). The working electrode (4 mm diameter) and the counter were printed with graphite ink, while the reference electrode was printed with silver paste. The size of the screen-printed electrode was: 3.4 × 1.0 × 0.05 cm (L × W × H).

## 3. Results and Discussion

### 3.1. Morphological Characterization of the Flexible Screen-Printed Electrodes (HP and HP-AuNPs)

**Figure 2:**
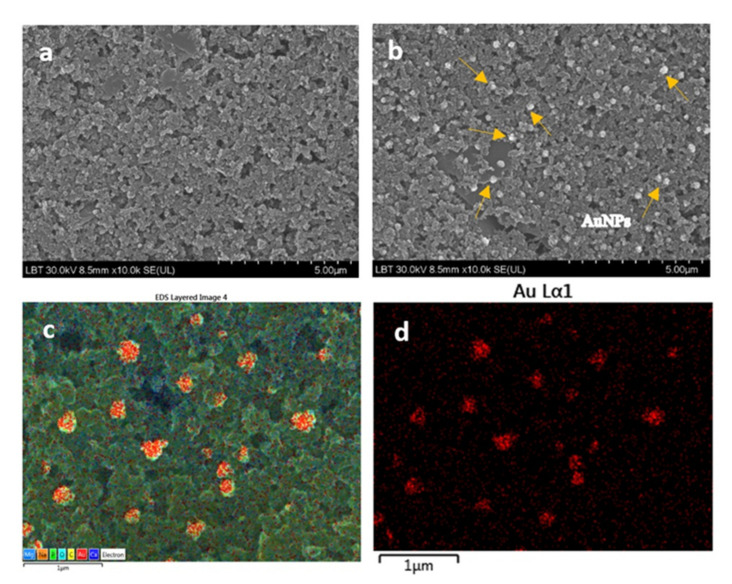
Scanning electron microscope (SEM) image of bare flexible screen-printed electrode (HP) (**a**) and gold nanoparticles (AuNPs)-modified electrode (HP-AuNPs) (**b**); scale bar: 5 µm. Energy-dispersive X-ray spectroscopy (EDS) images of HP-AuNPs electrode surface, confirming the presence of gold nanoparticles (**c**,**d**); scale bar: 1µm.

The surface of the new electrodes made on the stone paper substrate was morphologically characterized with SEM/EDS techniques. In Figure 2 are shown the SEM images for bare HP (a) and HP-AuNPs modified electrode (b–d). One can see that in the case of bare electrode the surface has a rough appearance. After chronoamperometric deposition of the AuNPs, particles with the mean size of 190 nm can be observed attached to the surface. The EDS analysis (Figure 2c,d) confirmed that the nanoparticles from the modified electrode surface were gold.

With the chronoamperometric method, we can customize the spatial distribution of AuNPs in terms of surface coverage. By increasing the electro-deposition time, more AuNPs are generated on the surface (see Figure S1a–c). However, there is a chance to fully cover the electrode surface with gold and so the electro catalytic effect is considerably decreased. The mean nanoparticle size varies from 150 nm to 385 nm, being strongly influenced by the electro-deposition time (see Figure S2 in Supplementary Materials).

The graphite powder employed to fabricate the screen-printed electrodes was characterized by various techniques, such as FTIR, Raman (see Figure S3) and BET (see the corresponding paragraph and Figure S4 in Supplementary Materials).

### 3.2. Electrochemical Characterization of the Flexible Screen-Printed Electrodes (HP and HP-AuNPs)

The HP and HP-AuNPs electrodes were electrochemically characterized by CV and EIS. Before testing the electrodes, they were cycled in a redox couple solution (1 mM K_4_[Fe(CN)_6_] + 0.2 M KCl), between −0.2…+0.65 V potential range (10 cycles; 50 mVs^−1^ scan rate).

**Figure 3:**
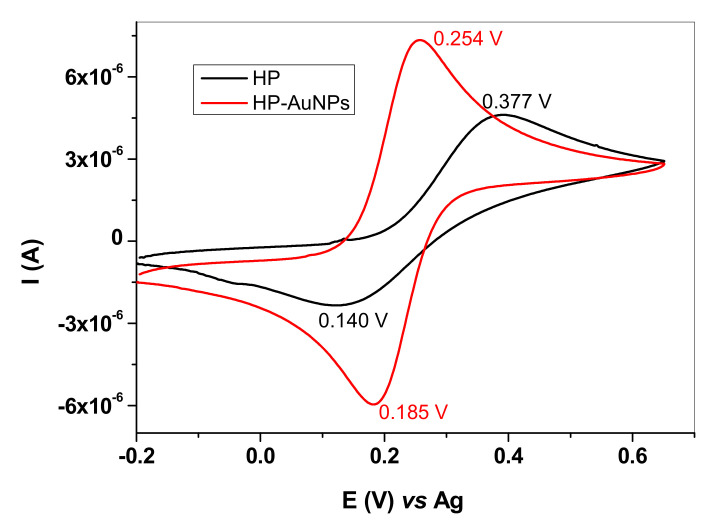
Cyclic voltammograms recorded with HP (black) and HP-AuNPs flexible electrode (red). Electrolyte: 1 mM K_4_[Fe(CN)_6_] + 0.2 M KCl; scan rate 10 mVs^−1^.

In Figure 3, the comparison between HP and HP-AuNPs electrode is shown. In the case of HP electrode, the peak potential separation (ΔE_p_) is very large (237 mV) and the oxidation/reduction waves are broad, indicating a quasi-reversible redox process. A different behavior can be observed for the HP-AuNPs electrode which exhibits considerably higher anodic/cathodic peak currents and smaller peak potential separation (~69 mV/n). Since the peak current ratio (Ip_a_/Ip_c_) is near 1 and the peak potential separation close to 60 mV/n, one can consider that HP-AuNPs electrode has remarkably improved redox characteristics [22].

In order to further characterize the two electrodes, their active areas were calculated using the Randles–Sevick Equation [23,24]:

*I_peak_* = ±2.687×10^5^AD^1/2^n^3/2^C*ν*^1/2^ (1)

where *I_peak_* represents the intensity of the anodic peak (A); D is the diffusion coefficient of K_4_[Fe(CN)_6_] (6.2 × 10^−6^ cm^2^/s); A is the active area (cm^2^) of bare or modified electrode; n is the number of electrons transferred during oxidation/reduction process; C is the concentration of [K_4_Fe(CN)_6_] in solution (mol/cm^3^) and *ν* is the scan rate (V/s).

**Figure 4:**
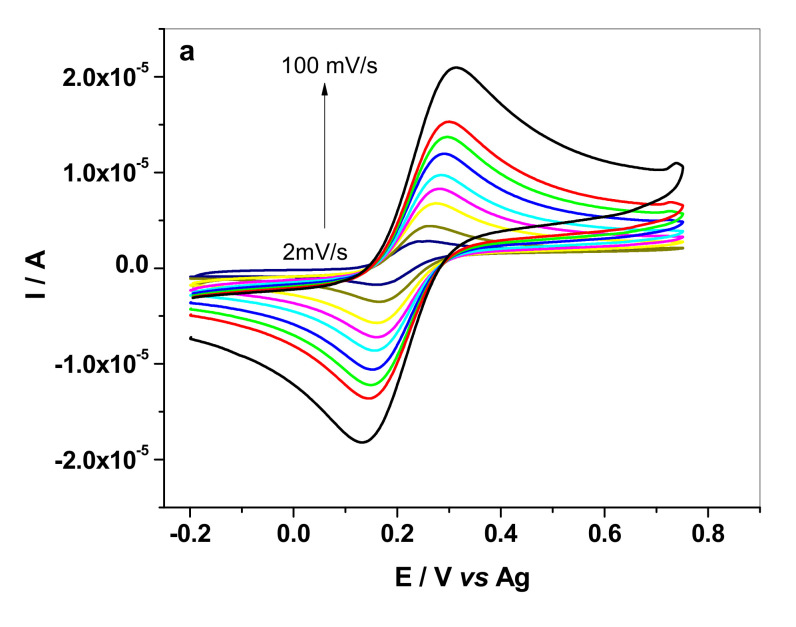

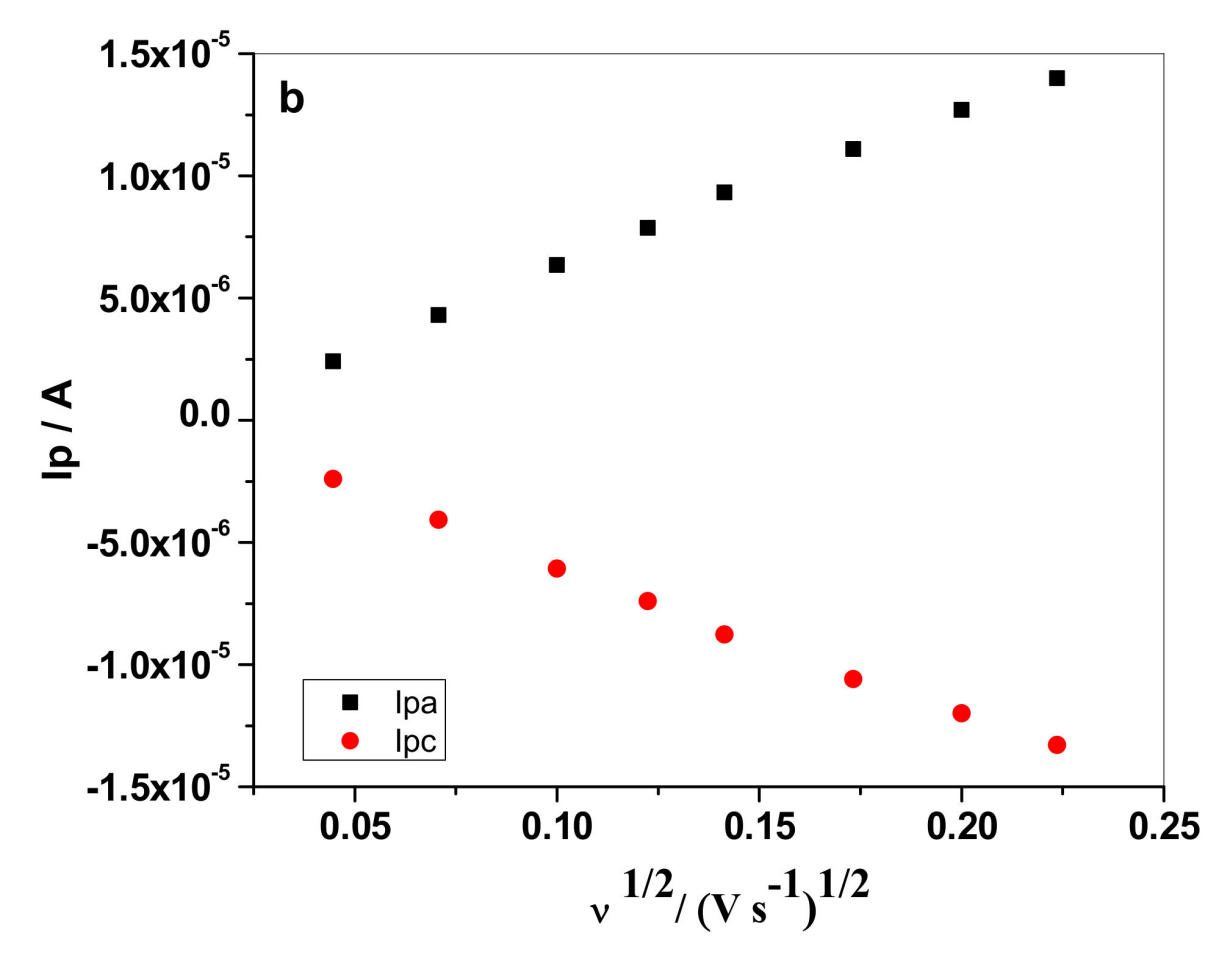
Cyclic voltammograms recorded with HP-AuNPs electrode in the presence of 1.0 mM K_4_[Fe(CN)_6_] + 0.2 M KCl, at different scanning rates, from 2 to 100 mVs^−1^ (2, 5, 10, 15, 20, 30, 40, 50, 100 mVs^−1^) (**a**). The linear plots obtained between anodic/cathodic peak current (I_p_) and the square root of scan rate (v^1/2^) (**b**).

For this purpose, cyclic voltammograms were recorded in solution containing 1 mM K_4_[Fe(CN)_6_] + 0.2 M KCl at different scanning rates, from 2 to 100 mVs^−1^ (see Figure 4a, for HP-AuNPs electrode).

The good linear correlation *(R^2^ = 0.9971)* obtained between the anodic/cathodic peak current (I_p_) and the square root of scan rate (v^1/2^) (Figure 4b) indicates that the redox process is diffusion-controlled. In addition, AuNPs led to a considerable increase of the active area (two times, as shown in Table 1). The formal potential (E^0′^) is also different for the bare and HP modified electrode (0.265 V for HP and 0.220 V for HP-AuNPs, at 10 mV·s^−1^) proving that the oxidation of the redox specie is highly favored in the case of AuNPs modified electrode. For the bare HP electrode, the CVs and the corresponding I_p_ vs. v^1/2^ plots can be seen in Figure S5, in Supplementary Materials.

**Figure 5:**
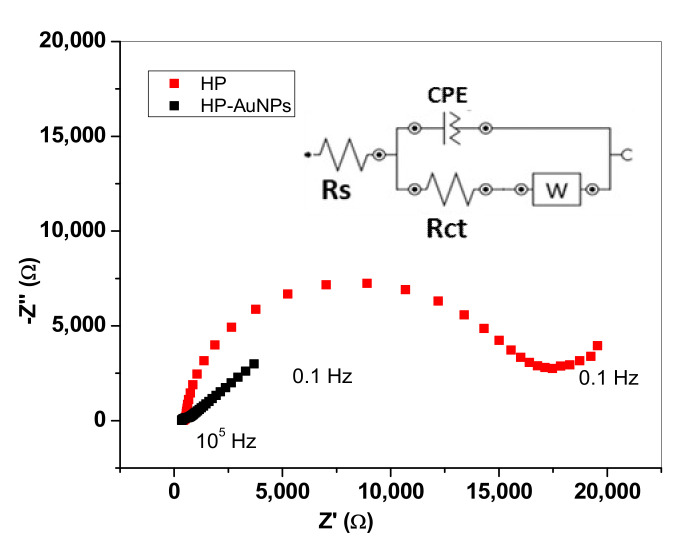
Nyquist plot obtained for HP (red) and HP-AuNPs (black) electrodes in solution containing 1.0 mM K_4_[Fe(CN)_6_] + 0.2 M KCl; applied potential: +0.265 V for HP and +0.220 V for HP-AuNPs.

The parameters obtained from CV measurements correlate well with other parameters obtained from EIS spectra, such as the charge-transfer resistance (R_ct_) and the apparent heterogeneous electron transfer rate constant (K_app_). Those parameters were obtained by fitting the Nyquist plots (Figure 5) with an equivalent electrical circuit (inset) that contains: the solution resistance (R_s_), the Warburg impedance (Z_W_), the charge-transfer resistance (R_ct_) and the constant phase element (CPE). CPE is generally used to replace the double-layer capacitance (C_dl_) and arises due to the roughness of the electrode surface [25].

In the case of bare HP electrode, the semicircle seen in the high-medium frequency range is due to the charge-transfer resistance, being remarkable high (15,700 Ω). For HP-AuNPs electrode its value is one order of magnitude lower (1220 Ω), proving the beneficial effect of AuNPs attached to the electrode surface.

The apparent heterogeneous electron transfer rate constant (K_app_) was also determined, due to its dependence on the charge-transfer resistance and the active area, as can be seen from Equation (2) [26]:

(2) $$K_{\mathrm{app}}=\frac{\mathrm{RT}}{n^{2}F^{2}{\mathrm{AR}}_{\mathrm{ct}}C}$$

where R is the ideal gas constant (8.314 Joule/(mol·K)); T is the temperature (298 K); F is the Faraday constant (96,485 C/mol); *n* is the number of electrons transferred during the redox reaction (n = 1); A is the active area of the electrode (cm^2^); R_ct_ is the charge-transfer resistance obtained from the fitted Nyquist plots (Ω); C is the concentration of the redox specie (mol/cm^3^).

For bare HP electrode, the value of K_app_ was found to be 2.8 × 10^−^^4^ cm/s while for HP-AuNPs it was one order of magnitude higher, of 1.94 × 10^−3^ cm/s (see Table 1). Based on the above results, one can conclude that the presence of AuNPs has a dual effect: it increases the active area of the HP electrode and highly promotes the transfer of electrons from the redox specie to the electrode surface.

### 3.3. Electrochemical Detection of Dopamine (DA) with HP and HP-AuNPs Flexible Electrodes

In order to investigate the analytical applicability of HP and HP-AuNPs electrodes, they were tested for dopamine detection in various electrolytes. The electrochemical measurements (CV and LSV) were recorded in pH 6 phosphate-buffered saline (PBS) solution, in artificial urine solution and in commercial solution containing dopamine (from Zentiva).

**Figure 6:**
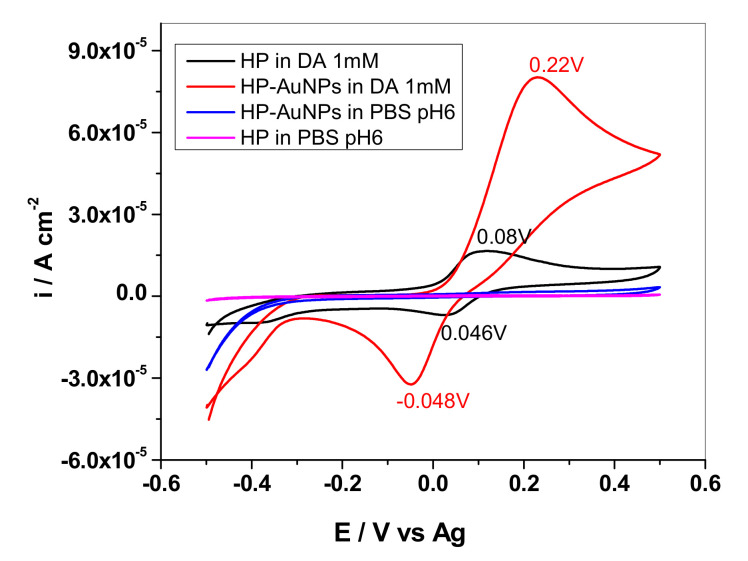
Cyclic voltammograms recorded with HP and HP-AuNPs flexible electrodes in the absence and presence of 1 mM dopamine. Supporting electrolyte: pH 6 phosphate-buffered saline (PBS); scan rate 10 mVs^−1^.

Cyclic voltammograms recorded with HP and HP-AuNPs electrodes in pH 6 PBS solution containing 1 mM dopamine are shown in Figure 6. One can see that there are major differences between the signals generated by the two electrodes. In the case of the bare HP electrode, the oxidation/reduction waves are considerably smaller than that corresponding to HP-AuNPs electrode (for a proper comparison of the signals, current densities are represented in this figure).

**Figure 7:**
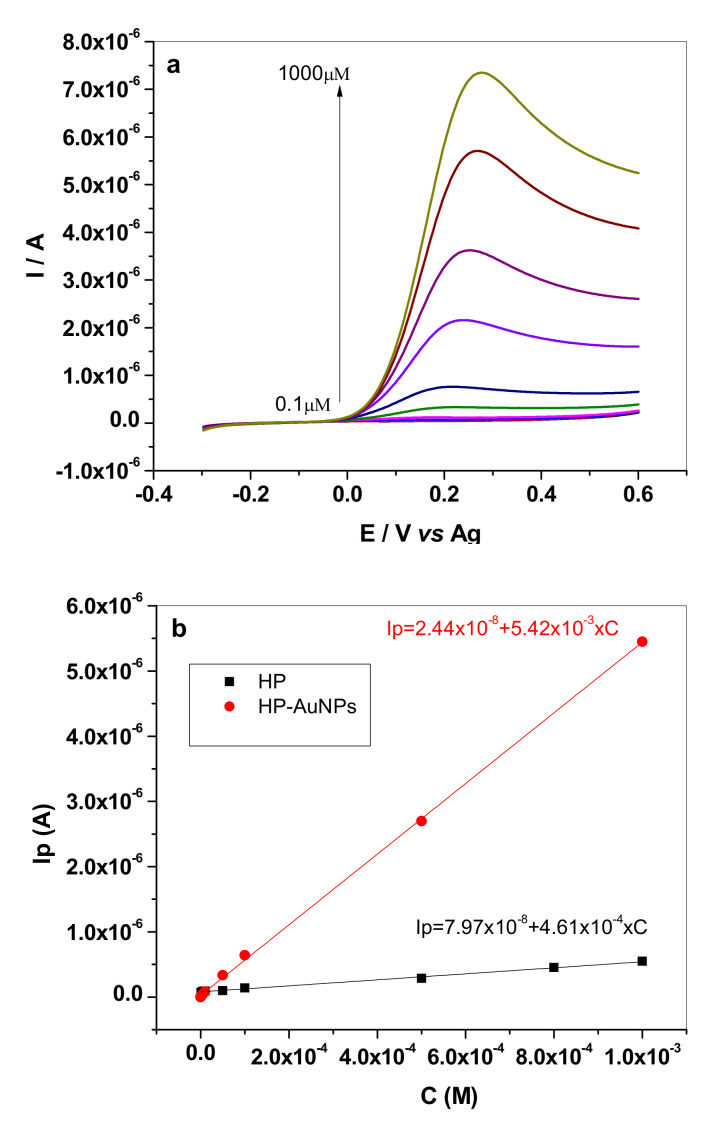
LSV recorded with HP-AuNPs electrode in pH 6 PBS solutions containing different concentrations of dopamine (10^−7^–10^−3^ M); scan rate 10 mVs^−1^ (**a**); the calibration curve for HP (10^−6^–10^−3^ M DA) and HP-AuNPs (10^−7^–10^−3^ M DA) electrode (**b**).

The results are further confirmed by the calibration plots obtained with the two electrodes. For example, in Figure 7a are shown the LSVs recorded with HP-AuNPs in pH 6 PBS solutions containing different concentrations of dopamine (10^−7^–10^−3^ M). The calibration plots for HP (10^−6^–10^−3^ M DA) and HP-AuNPs (10^−7^–10^−3^ M DA) electrodes are represented in Figure 7b, where the higher sensitivity of the modified electrode (5.42 mA/M) in comparison with bare electrode (0.46 mA/M) can be clearly observed.

The analytical performances of the tested electrodes in pH6 PBS were compared with those of other electrodes from the literature and are listed in Table 2. As expected, HP-AuNPs electrode has a very wide linear range (three decades) and a low detection limit (0.03 µM) comparable with that of other types of modified electrodes. In addition, the limit of quantification (LOQ = 1 × 10^−7^ M) was considerable smaller than that of the bare HP electrode (1 × 10^−6^ M).

**Figure 8:**
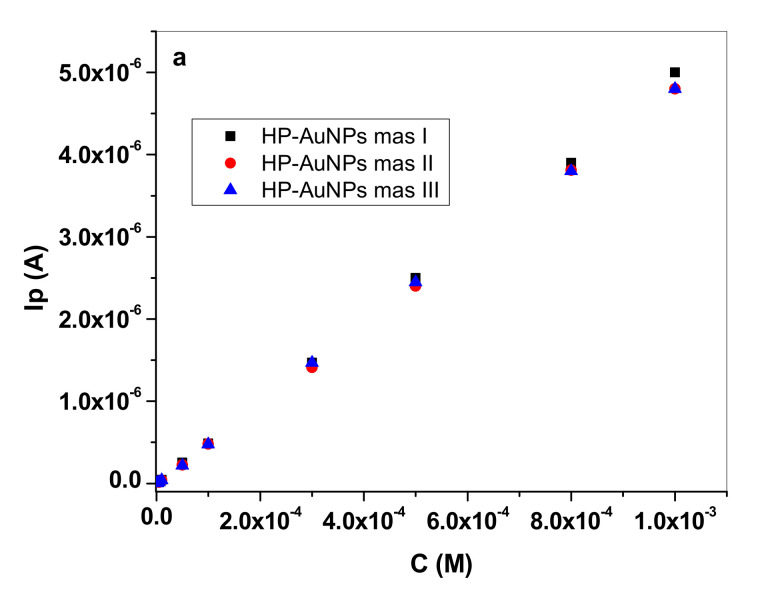

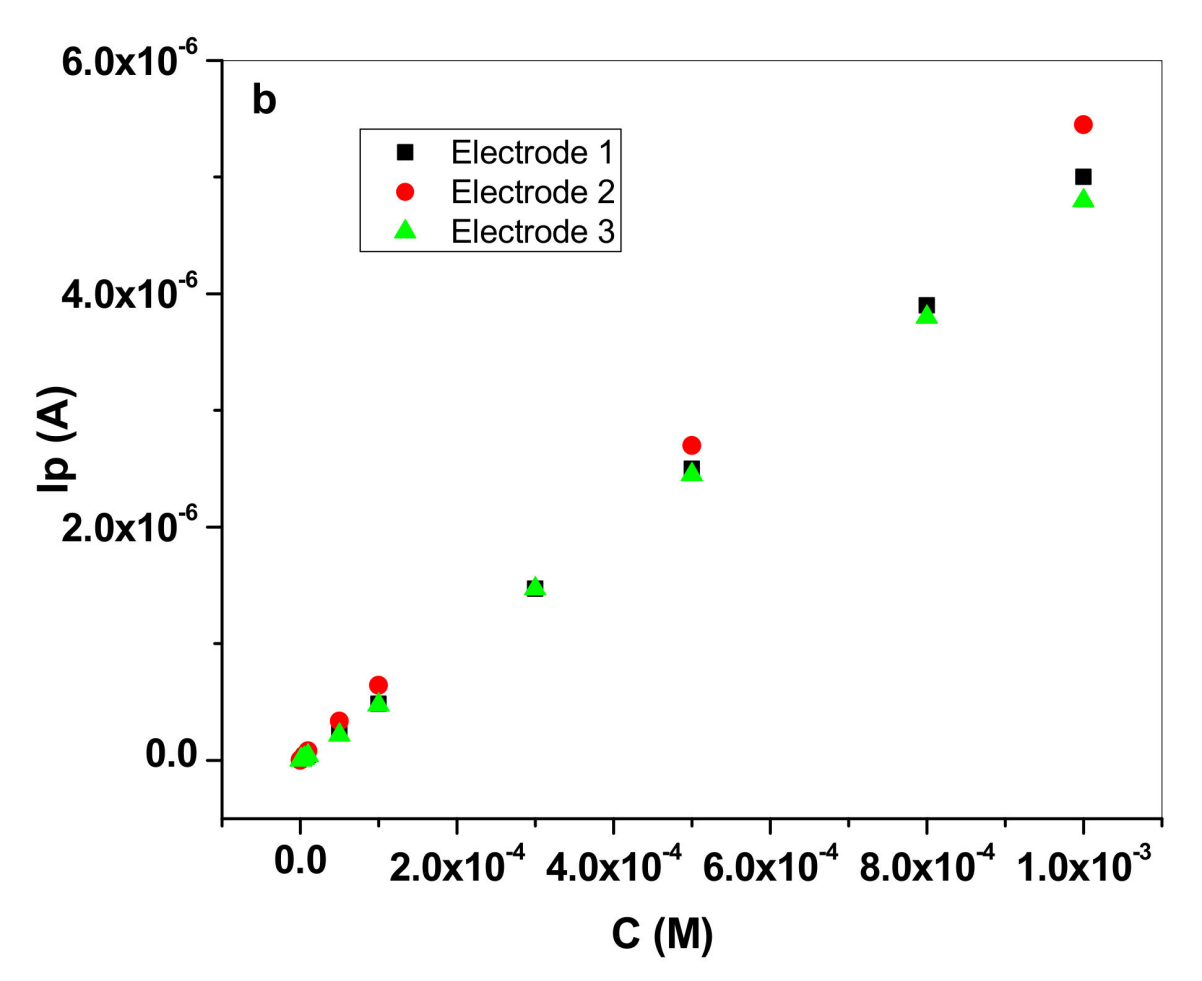
Calibration curves obtained with HP-AuNPs electrode in pH 6 PBS solutions containing different concentrations of dopamine (10^−7^–10^−3^ M), for three consecutive measurements performed with the same (**a**) and different electrodes (**b**).

Since the HP-AuNPs electrode had superior performances in terms of linear range and limit of detection in comparison with the HP electrode, other important characteristics such as repeatability and reproducibility were investigated. In Figure 8a are shown three consecutive measurements obtained with the same HP-AuNPs electrode. After each complete series of measurements the electrode was thoroughly washed with distilled water then cycled in pH 6 PBS solution, until no signal from dopamine was recorded. As can be seen, the repeatability of the modified electrode was excellent, with Relative Standard Deviation (RSD) of 2.04% for 0.5 mM DA concentration (n = 3). The reproducibility was also tested by fabricating three electrodes with the same procedure and the recorded signals, plotted in Figure 8b, were also very close. In this case, the RSD value was 5.18% (n = 3) for 0.5 mM DA concentration.

The time stability of HP-AuNPs electrode was also investigated by recording the DA signal (10^−4^ M in pH 6 PBS) during one month. As can be seen in Figure S6 (Supplementary Materials) the signal slightly decreased (95%) after two weeks then remained stable, proving the applicability of such electrodes in electrochemical sensing devices.

For real samples analysis it was necessary to get a new calibration curve in a medium more relevant to clinical analysis (e.g., artificial urine). The choice of this medium was based on the fact that the preservation of urine samples is generally simple, and the clinical analysis may give us information about the level of DA in the body and the health status.

**Figure 9:**
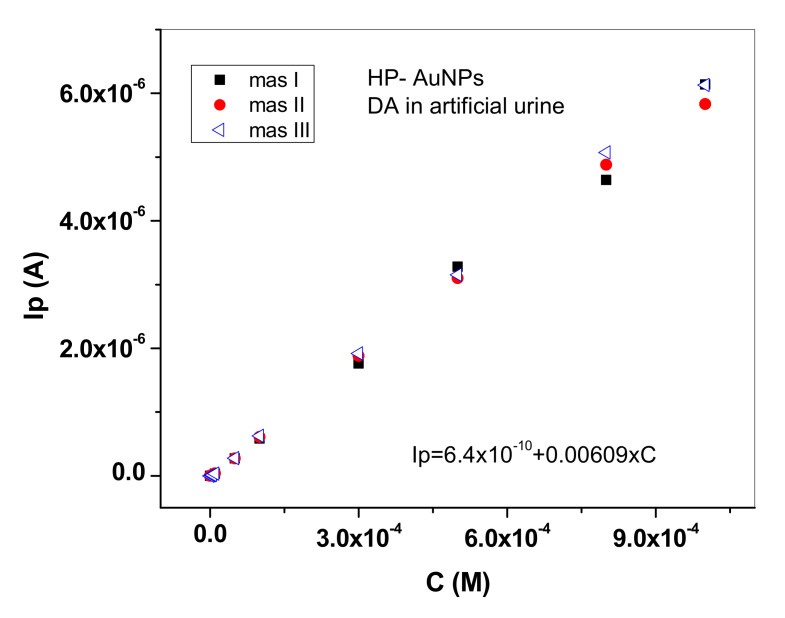
Calibration curves obtained with HP-AuNPs electrode in artificial urine solutions containing different concentrations of dopamine (10^−7^−10^−3^ M); the three consecutive measurements were performed with the same electrode (RSD is 2.92% for 0.5 mM DA concentration).

In Figure 9 are represented three successive calibration curves obtained in artificial urine with the same HP-AuNPs electrode. By comparison with the calibration plot obtained in pH 6 PBS solution (Figure 7b), the sensitivity value is higher in artificial urine (6.09 mA/M) possibly due the complex composition of the matrix, a fact also reported in a recent paper [9].

**Figure 10:**
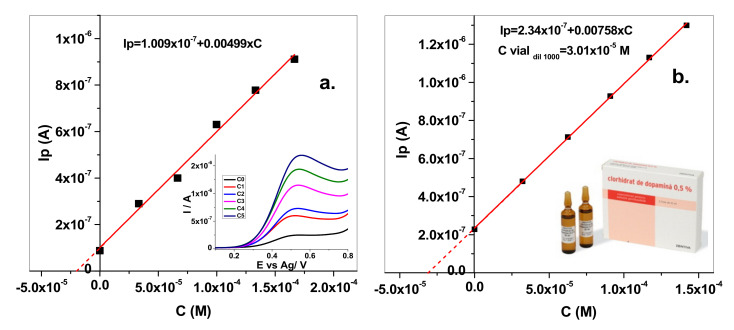
Standard addition method for the determination of DA concentration in artificial urine (**a**) and pharmaceutical drug solution (**b**).

The determination of DA was then tested in spiked artificial urine and in a pharmaceutical drug solution, from ZENTIVA that contained dopamine hydrochloride (5 mg/mL) (Figure 10a,b, respectively). Hence, the method was applied for DA determination in an artificial urine solution that contained an unknown concentration of dopamine (C_o_). In the same solution, known volumes of dopamine from a standard solution were added and the corresponding calibration curve was obtained (Figure 10a; inset: the LSV signal recorded for each concentration). The added/found dopamine concentration value can be seen in Table 3, both for artificial urine and the pharmaceutical drug solution. The results indicate a very good DA determination in artificial urine, without significant matrix effects. In the case of the pharmaceutical drug solution, the DA determination was affected by the interfering species present in solution: sodium metabisulfite (Na_2_S_2_O_5_), maleic acid (C_4_H_4_O_4_), sodium chloride (NaCl), and propylene glycol (C_3_H_8_O_2_).

## 4. Conclusions

Flexible electrodes printed on stone paper and modified with gold nanoparticles (HP-AuNPs) were fabricated and morphologically investigated by SEM/EDS to confirm the attachment of AuNPs to the electrode surface. Their electrochemical properties were studied by Cyclic Voltammetry (CV) and Electrochemical Impedance Spectroscopy (EIS) and the results indicated the beneficial effect of AuNPs: a lower charge-transfer resistance and higher apparent heterogeneous electron transfer rate constant, in comparison with the bare electrode (HP). In addition, the resulting modified electrode showed excellent electro-catalytic activity towards dopamine, being successfully used for the accurate determination of dopamine in complex samples.

## Figures and Tables

**Table 1:** The electrochemical parameters of flexible screen-printed electrodes, HP and HP-AuNPs.

Electrode	ΔEp (mV)	I_pa_/I_pc_	A (cm^2^)	E^0′^ (V)	R_ct_ (Ω)	K_app_ (cm/s)
HP	237	1.3	0.06	0.265	15,700	2.8 × 10^−4^
HP-AuNPs	69	1.0	0.112	0.220	1220	1.94 × 10^−3^

**Table 2:** Analytical performances of HP and HP-AuNPs electrodes for dopamine (DA) detection compared with other types of electrode.

Electrode	Substrate	Technique	LOD (µM)	Linear Domain (M)	Ref.
HP	Stone paper	LSV	0.3	10^−6^–10^−3^	This work
HP-AuNPs	Stone paper	LSV	0.03	10^−7^–10^−3^	This work
SPCE	PET	SWV	0.73	5 × 10^−6^–10^−5^	[1]
e-FGPE	graphite paper	DPV	0.01	0.5 × 10^−6^–35 × 10^−6^	[16]
SPE-MWCNT	ceramic	DPV	0.015	5·× 10^−8^–10^−6^	[17]
AuNPs-SPC	Polystyrene based film	SWV	0.008	2·× 10^−7^–10^−4^	[19]
SPCE/CB-ERGO	PET	SWV	0.41	10^−6^–10^−5^	[27]
Pt-AuNPs/LIG/PDMS	PDMS	DPV	0.075	9.5 × 10^−7^–3 × 10^−5^	[28]
Graphene/AuNP/GCE	GCE	DPV	1.86	5 × 10^−6^–10^−3^	[29]

PET—polyethylene terephthalate; PDMS—polydimethylsiloxane; LIG—laser-induced graphene; e-FGPE—exfoliated flexible graphite paper electrode; CB-ERGO—carbon black-electrochemically reduced graphene oxide; SPCE—screen-printed carbon electrode; SPC—screen-printed carbon; SPE-MWCNT—screen-printed electrode- multi-wall carbon nanotube; GCE—glassy carbon electrode; LSV—linear sweep voltammetry; SWV—square wave voltammetry; DPV—differential pulse voltammetry.

**Table 3:** Determination of DA concentration in artificial urine and pharmaceutical drug solution.

Solution	Added (M)	Found (M)	Recovery (%)	RSD (%)
Artificial urine	2 × 10^−5^	2.02 × 10^−5^	101	3.5
Pharmaceutical drug solution	2.63 × 10^−5^	3.01 × 10^−5^	114	6.5

## Supplementary Materials

The following are available online at https://www.mdpi.com/1424-8220/20/12/3609/s1, Figure S1. Scanning electron microscope (SEM) images of flexible screen-printed electrodes modified with gold nanoparticles (HP-AuNPs) showing the surface coverage after different electro-deposition times: 60 s (a); 120 s (b); 240 s (c); scale bar: 1 µm., Figure S2. Histograms showing the particle size distribution at various electro-deposition times: 60 s; 120 s and 240 s., Figure S3. Fourier transform infrared (FTIR) (a) and Raman (b) spectrum of graphite powder., Figure S4. N2 adsorption–desorption isotherms (a) and pore size distribution for the graphite powder (b)., Figure S5. Cyclic voltammograms recorded with HP electrode in the presence of 1.0 mM K4[Fe(CN)6] + 0.2 M KCl, at different scanning rates, from 2 to 100 mVs−1 (2, 5, 10, 15, 20, 30, 40, 50, 100 mVs−1) (a). The linear plots obtained between anodic/cathodic peak current (Ip) and the square root of scan rate (v1/2) (b)., Figure S6. The time stability of HP-AuNPs electrode investigated during 30 days; 10-4 M dopamine (DA) in pH 6 phosphate-buffered saline (PBS).
