# Supplementary material for: Stone Paper as a New Substrate to Fabricate Flexible Screen-Printed Electrodes for the Electrochemical Detection of Dopamine
Source: Sensors (Basel). 2020 Jun 26;20(12):3609. doi: 10.3390/s20123609 (PMC7349771; doi:10.3390/s20123609)
Supplement: Supplementary file 1 [file sensors-20-03609-s001.pdf]

Stone Paper as a New Substrate to Fabricate Flexible Screen-Printed Electrodes for the Electrochemical Detection of Dopamine

Codruta Varodi [1], Florina Pogacean [1], Marin Gheorghe [2], Valentin Mirel [1], Maria Coros [1], Lucian Barbu-Tudoran [1], Raluca-Ioana Stefan-van Staden [3,4] and Stela Pruneanu [1,*]

[1] National Institute for Research and Development of Isotopic and Molecular Technologies, Donat Street, No. 67–103, RO, 400293, Cluj-Napoca, Romania, codruta.varodi@itim-cj.ro (C.V.); florina.pogacean@itim-cj.ro (F.P.); valentin.mirel@itim-cj.ro (V.M.); maria.coros@itim-cj.ro (M.C.); lucianbarbu@yahoo.com (L.B.T.)

[2] NANOM MEMS srl, G. Cosbuc Street, No. 9, RO, 505400, Rasnov, Brasov, Romania, maringhe@nanom-mems.com (M.G.);

[3] Laboratory of Electrochemistry and PATLAB, National Institute of Research for Electrochemistry and Condensed Matter, 202 Splaiul Independentei Street, 060021, Bucharest-6, Romania, ralucavanstaden@gmail.com (R.I.S.V.S.)

[4] Faculty of Applied Chemistry and Material Science, Politehnica University of Bucharest, Bucharest 060042, Romania

*Correspondence: stela.pruneanu@itim-cj.ro

Received: 25 May 2020; Accepted: 23 June 2020; Published: date

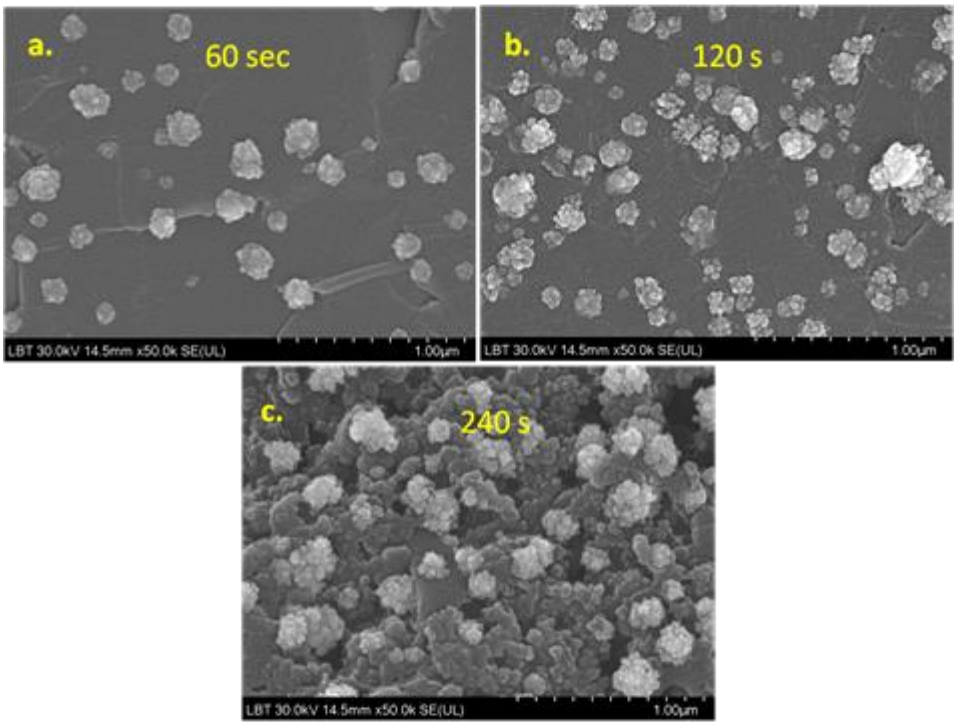

Figure S1. Scanning electron microscope (SEM) images of flexible screen-printed electrodes modified with gold nanoparticles (HP-AuNPs) showing the surface coverage after different electro-deposition times: 60 s (**a**); 120 s (**b**); 240 s (**c**); scale bar: 1 μm.

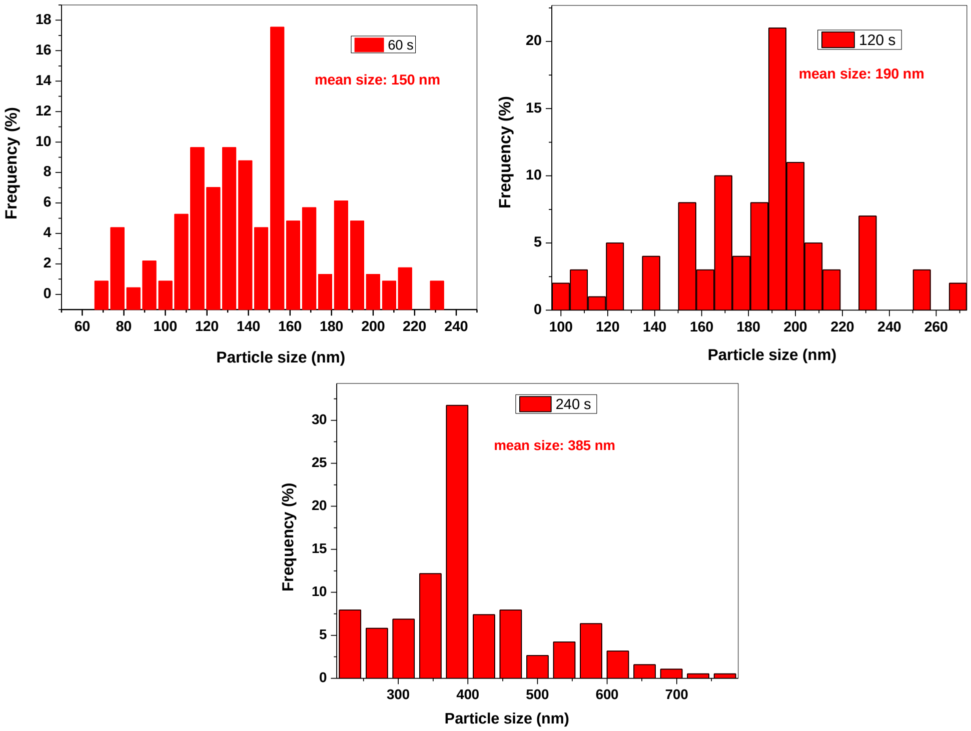

Figure S2. Histograms showing the particle size distribution at various electro-deposition times: 60 s; 120 s and 240 s.

The graphite powder employed to fabricate the screen-printed electrodes was characterized by various techniques, such as Fourier transform infrared (FTIR) spectroscopy, Raman and Brunauer–Emmett–Teller (BET). The FTIR spectrum of pure graphite (Figure S3a) exhibits an intense peak at 3452 cm^{-1}, which is associated to the bending modes of water molecules. The intense peak at 1640 cm^{-1} is ascribed to the existence of C=C bond in pure graphite powder, the peak from 1389 cm^{-1} correspond to C–C stretch from the structure and the peak from 1100 cm^{-1} is associated with the =C–H bending mode.

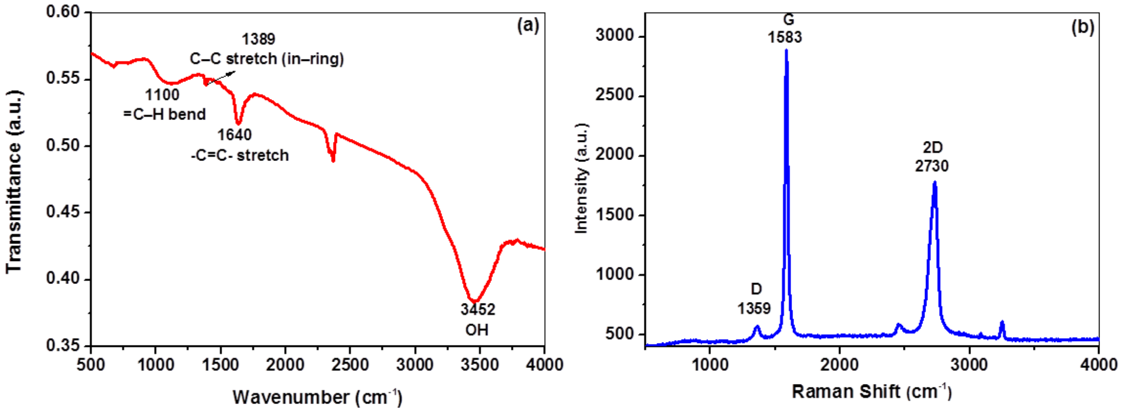

Figure S3. Fourier transform infrared (FTIR) (**a**) and Raman (**b**) spectrum of graphite powder.

In the Raman spectrum of graphite (Figure S3b) two intense peaks can be seen at 1583 cm^{-1} (G band) and 2730 cm^{-1} (2D band). The first peak corresponds to the degenerate in-plane E2g optical mode at the center of the Brillouin zone, while the second peak corresponds to the second order Raman scattering of the in-plane transverse optical mode, close to the zone boundary K point [1]. The weak D band at 1359 cm^{-1} appears in disordered carbon materials, and its intensity is related to the presence of six-fold aromatic rings. The intense 2D band is generally used to characterize and estimate the crystallinity of graphite.

Next, the N_2 adsorption–desorption isotherms of the powder graphite were recorded (Figure S4a). The isotherms are of type IV, with a small hysteresis loop on the desorption branch, associated with mesoporous structure [2]. Total specific surface area of 8.03 m^2/g for the graphite powder was calculated in the 0.01 < p/p0 < 0.30 interval, using the BET model. Porosity of the sample was estimated from the desorption branch using the Dollimer–Heal model. Thus, specific pore volume of 0.022 cm^3/g was calculated, while pores have diameters between 2 and 10 nm (Figure S4b).

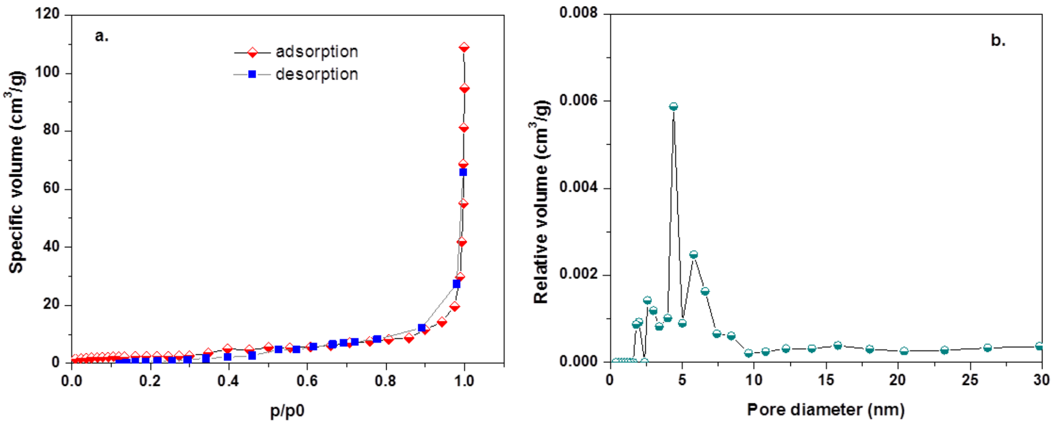

Figure S4. N_2 adsorption–desorption isotherms (**a**) and pore size distribution for the graphite powder (**b**).

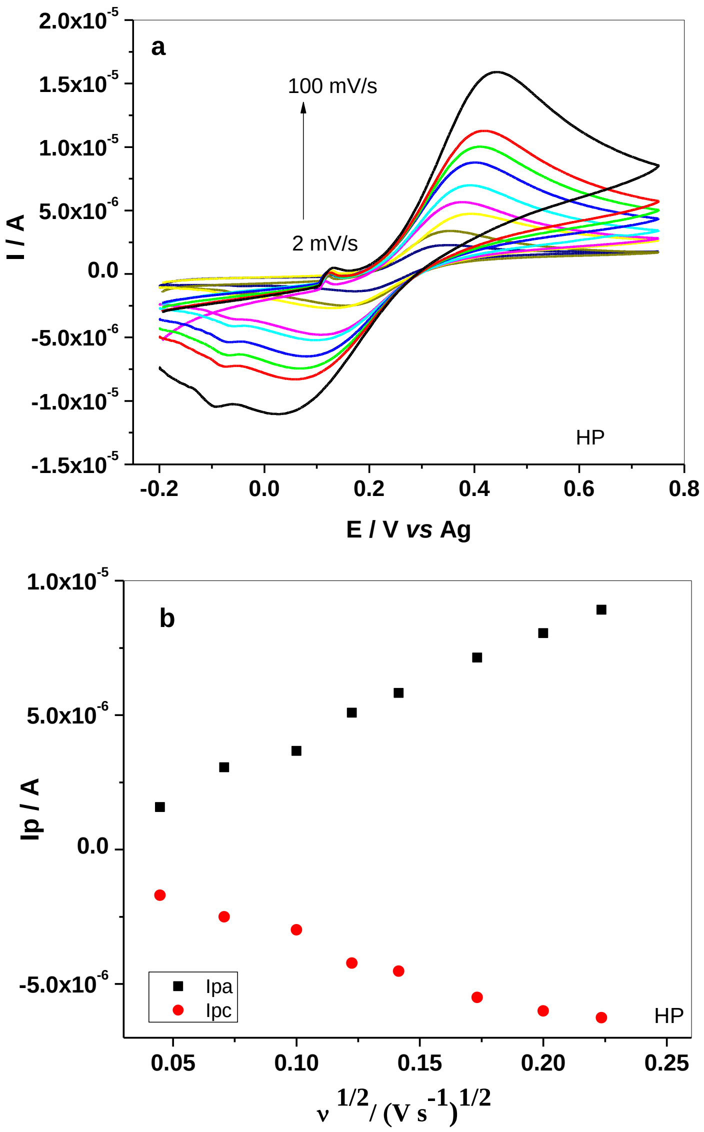

Figure S5. Cyclic voltammograms recorded with HP electrode in the presence of 1.0 mM $K_4[Fe(CN)_6]$ + 0.2 M KCl, at different scanning rates, from 2 to 100 mVs^{-1} (2, 5, 10, 15, 20, 30, 40, 50, 100 mVs^{-1}) (**a**). The linear plots obtained between anodic/cathodic peak current (I_p) and the square root of scan rate ($v^{1/2}$) (**b**).

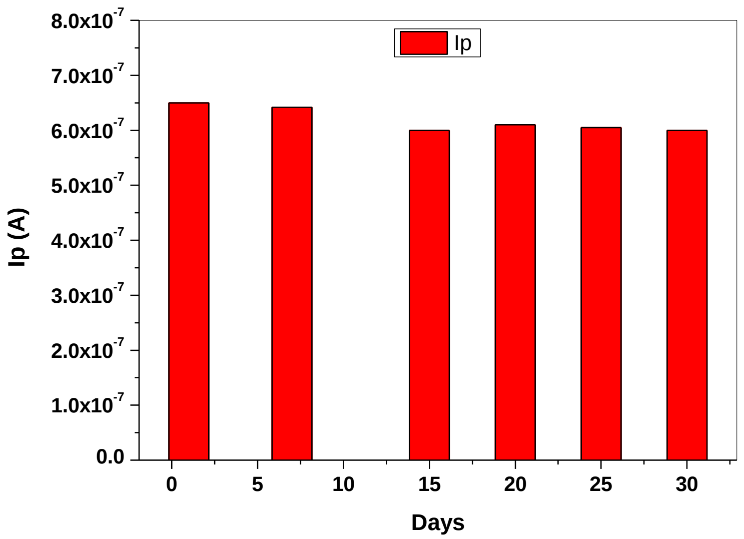

Figure S6. The time stability of HP-AuNPs electrode investigated during 30 days; 10^{-4} M dopamine (DA) in pH 6 phosphate-buffered saline (PBS).

The RSD (%) was calculated according to formula: RSD = $100 \cdot S/X_{mean}$, where X_{mean} is the mean value of the recorded signals (from 3 measurements) and S is the standard deviation of the signal (from 3 measurements).

The limit of quantification (LOQ) is the lowest concentration at which the electrode generates a signal. The limit of quantification of HP-AuNPs electrode (LOQ = 1×10^{-7} M) was considerably smaller than that of the bare HP electrode (1×10^{-6} M).

The limit of detection (LOD) was calculated with the formula: LOD = LOQ/3.3.

References

1. Jorio, A. Raman spectroscopy in graphene-based systems: Prototypes for nanoscience and nanometrology. *Int. Sch. Res. Netw.* **2012**, doi:10.5402/2012/234216
2. K. Sing, D. Everett, R. Haul, L. Moscou, R. Pierotti, J. Rouquerol, T. Siemienewska, *Pure Appl. Chem.* **1985**, *57*, 603–619.
